# Association of gut microbiome and metabolites with onset and treatment response of patients with pemphigus vulgaris

**DOI:** 10.3389/fimmu.2023.1114586

**Published:** 2023-04-14

**Authors:** Yiyi Wang, Xuyang Xia, Xingli Zhou, Tongying Zhan, Qinghong Dai, Yan Zhang, Wei Zhang, Yang Shu, Wei Li, Heng Xu

**Affiliations:** ^1^ Department of Dermatology & Rare Disease Center, West China Hospital, Sichuan University, Chengdu, China; ^2^ State Key Laboratory of Biotherapy and Cancer Center, West China Hospital, Chengdu, China; ^3^ Department of Clinical Pharmacology, Hunan Key Laboratory of Pharmacogenetics, Xiangya Hospital, Central South University, Changsha, China; ^4^ Lung Cancer Center, West China Hospital, Sichuan University, Chengdu, China; ^5^ Department of Laboratory Medicine, Research Center of Clinical Laboratory Medicine, West China Hospital, Sichuan University, Chengdu, China

**Keywords:** pemphigus vulgaris, microbiome, glucocorticoid, short-chain fatty acids, metagemonic

## Abstract

**Background:**

Gut dysbiosis and gut microbiome-derived metabolites have been implicated in both disease onset and treatment response, but this has been rarely demonstrated in pemphigus vulgaris (PV). Here, we aim to systematically characterize the gut microbiome to assess the specific microbial species and metabolites associated with PV.

**Methods:**

We enrolled 60 PV patients and 19 matched healthy family members, and collected 100 fecal samples (60 treatment-naïve, 21 matched post-treatment, and 19 controls). Metagenomic shotgun sequencing and subsequent quality control/alignment/annotation were performed to assess the composition and microbial species, in order to establish the association between gut microbiome with PV onset and treatment response. In addition, we evaluated short-chain fatty acids (SCFAs) in PV patients through targeted metabolomics analysis.

**Results:**

The diversity of the gut microbiome in PV patients deviates from the healthy family members but not between responder and non-responder, or before and after glucocorticoid treatment. However, the relative abundance of several microbial species, including the pathogenic bacteria (e.g., *Escherichia coli*) and some SCFA-producing probiotics (e.g., *Eubacterium ventriosum*), consistently differed between the two groups in each comparison. *Escherichia coli* was enriched in PV patients and significantly decreased after treatment in responders. In contrast, *Eubacterium ventriosum* was enriched in healthy family members and significantly increased particularly in responders after treatment. Consistently, several gut microbiome-derived SCFAs were enriched in healthy family members and significantly increased after treatment (e.g., butyric acid and valeric acid).

**Conclusions:**

This study supports the association between the gut microbiome and PV onset, possibly through disrupting the balance of gut pathogenic bacteria and probiotics and influencing the level of gut microbiome-derived SCFAs. Furthermore, we revealed the potential relationship between specific microbial species and glucocorticoid treatment.

## Introduction

Pemphigus is a severe, recurrent, potentially fatal autoimmune bullous disease characterized by the formation of flaccid blisters and erosive lesions on the skin or mucous membranes (1). Multiple pemphigus subtypes were identified, with pemphigus vulgaris (PV) ranking at the top in terms of incidence. The production of pathogenic autoantibodies against the desmosomal adhesion glycoproteins desmoglein (Dsg)1 and Dsg3 is considered the direct cause of pemphigus (1–3). Nevertheless, the molecular mechanisms underlying the onset of pemphigus remain poorly understood. Diverse risk factors have been explored, including several HLA haplotypes as inherited predispositions and environmental factors such as adverse drug reactions, viral infections, diet, and stress (1, 2, 4–6)

Environmental factors are believed to be key determinants of gut microbial composition and function, and dysbiosis of the gut microbiome can induce local and systemic immune responses in the host, thereby influencing the onset of autoimmune diseases (7). Indeed, numerous studies have identified significant differences in the gut microbiome between healthy controls and patients with autoimmune diseases (e.g., rheumatoid arthritis, systemic lupus erythematosus, and ankylosing spondylitis) (8–10). Their immunologic process might be influenced by the disturbed gut microbiome through several hypothesized pathways, including oxidative phosphorylation and biosynthesis of branched-chain amino acids (8, 11, 12). Similarly, the association between the gut microbiome and PV onset has also been revealed (13), which suggested a few specific PV-associated microbial species due to the limitation of sample size and 16S rRNA sequencing technical. Therefore, larger sample sizes and higher-resolution approaches are required for a more comprehensive analysis. In contrast to 16S rRNA sequencing, metagenomic sequencing can locate the differential bacteria at the species level and obtain more comprehensive information on detailed functional genes and pathways (14–16).

Moreover, complex bidirectional interactions between the gut microbiome and drugs/treatment outcomes were also revealed (17). Multiple non-antibiotic drugs can alter the composition viability of the gut microbiome, while the gut microbiome may in turn regulate the therapeutic effects and toxicities of drugs (17–22). Recently, *in vitro* screening of the extensive impact of non-antibiotic drugs on human gut bacteria has revealed that approximately a quarter of the marketed non-antibiotic drugs exhibit an inhibitory effect on the representative gut microbial strains (23). Not surprisingly, the association between the gut microbiome and individualized drug response (e.g., anti-TNFα) has been investigated in autoimmune diseases (24–26). For PV treatment, although most patients remit and recover after receiving typical conventional treatment, some cases remain refractory and resistant to conventional therapy, which may be attributed to the individualized microbiome.

Gut microbiome-derived metabolites are small molecules produced as intermediate or final products of microbial metabolism. They are one of the primary mechanisms by which the gut microbiome interacts with the host, and exhibit an important and diverse effect on host physiology (27, 28). Specific classes of gut microbiome-derived metabolites, such as short-chain fatty acids (SCFAs), have been implicated to act on numerous cell types to regulate many biological processes, including host metabolism and immune function (29), thus contributing to the onset of diseases and treatment response (30–32). It is worthwhile to investigate whether gut microbiome-derived metabolites play a role in the underlying mechanism of PV.

In this study, we systematically explored the gut microbiome and metabolites in PV patients through metagenomic shotgun sequencing and a targeted metabolomics approach, shedding light on their potential roles in PV onset and treatment response.

## Materials and methods

### Study cohort and patient information

Patients in this study were derived from the autoimmune blister disease cohort in the Department of Dermatology, West China Hospital of Sichuan University. All samples were collected from November 2017 to April 2019. The study was approved by the local ethics committee [West China Hospital, Sichuan University, approval no. 2017 (241)], and all participants signed informed consent forms.

The PV group comprised glucocorticoid-naïve patients with clinically confirmed PV between the ages of 18 and 80 years. PV was diagnosed strictly by the accepted standard (10): patients must have typical clinical manifestations of pemphigus vulgaris, intraepidermal blistering and acantholysis on histopathology, net-like immunoglobulin G (IgG), and complement component 3 (C3) deposition on mucosa and/or skin membranes detected by direct immunofluorescence and increased anti-Dsg3 antibody with or without anti-Dsg1 antibody in serum. Controls were healthy family members of some patients in the PV group who were free of skin diseases and live together with their matched pemphigus patients. The healthy controls also have similar eating habits, lifestyles, and living environments with PV patients and are generally comparable to PV patients in terms of sex, age, and body mass index (BMI) (**Table S1**). Individuals were excluded if they had a medication history within three months before sampling (including antibiotics, probiotics, immunosuppressors, etc.) or any other diseases (including other autoimmune diseases, metabolic diseases, malignant tumors, visceral organ dysfunction, etc.). Pregnant or lactating women were also excluded.

Detailed demographic and clinical information of all subjects was collected and presented in **Table S1**, including sex, age, BMI, affected skin/mucosa, pemphigus disease area index (PDAI), anti-Dsg1 antibody titers, and anti-Dsg3 antibody titers. PDAI was evaluated by two experienced dermatologists, while laboratory indexes were measured by the Department of Laboratory Medicine in our hospital according to standard procedures.

At the time of sampling, PDAI scores were evaluated for patients in the PV group. Subsequently, following the British guidelines for the management of pemphigus (33), the certain treatment option was applied for one patient based on disease severity (mild, moderate, or severe), disease stage (acute progressive or stable stage), relative restriction of large-dose GC, etc. In this cohort, 46 of them received glucocorticoid-alone therapy and 14 of them who are not suitable for large-dose glucocorticoid (e.g., elder patients) or at disease acute progressive stage received glucocorticoid combined with azathioprine/cyclosporine therapy. The PDAI scores were reassessed after one month. PDAI improvement rate (ΔPDAI) referred to the percentage of reduction in PDAI scores after one month of conventional treatment. Patients were defined as the responder group if their ΔPDAI were more than or equal to 50%, whereas refractory pemphigus vulgaris, namely the non-responder group, had PDAI scores that decreased by less than 50%. Furthermore, after one month of glucocorticoid-alone therapy, 21 patients were resampled and separated into the responder and non-responder groups to explore the gut microbial changes after glucocorticoid treatment.

### Sample collection and DNA extraction

Fresh stool samples were collected in the hospital and then immediately transported to the laboratory in an ice bag. In the laboratory, the samples were divided into four 15 mL centrifuge tubes containing 1 g of stool each and stored at -80°C for further processing. DNA extraction was performed using the CTAB method. The DNA concentration was measured using a Qubit^®^ dsDNA Assay Kit in a Qubit^®^ 2.0 fluorometer, and its degradation degree was monitored on 1% agarose gels.

### DNA library construction and sequencing

A total amount of 1 μg DNA per sample was used as input material for the DNA sample preparations. Utilizing the NEBNext^®^ UltraTM DNA Library Prep Kit for Illumina (NEB, USA) following the manufacturer’s instructions, sequencing libraries were created, and index codes were added to assign sequences to specific samples. Briefly, after being sonicated to a fragment size of 350 bp, the DNA sample was end-polished, A-tailed, and ligated with the full-length adaptor for Illumina sequencing with further PCR amplification. Finally, libraries were evaluated for size distribution using an Agilent2100 Bioanalyzer, and quantities were determined using real-time PCR after PCR products had been purified (AMPure XP system). On a cBot Cluster Generation System, the index-coded samples were clustered in accordance with the manufacturer’s recommendations. Following cluster creation, the library preparations were sequenced on an Illumina HiSeq platform, resulting in the production of paired-end reads.

### Gene catalog construction

Following quality control, the sequencing reads were *de novo* assembled into contigs using Megahit v1.2.9 (11). Gene prediction from the assembled contigs was performed using Prokka v1.13 (12). Using CD-hit (13), redundant genes with 90% coverage and 95% similarity were eliminated. Finally, we used Salmon v1.4.0 (14) to quantify the relative abundances of the genes and obtained a nonredundant gene catalog comprising 4045593 genes.

### Taxonomical annotation

For the accuracy of taxonomical annotation, we used MetaPhlAn2 (15) for alignment and annotation based on bacterial marker genes. After obtaining a taxonomical relative abundance profile, we used the Galaxy online platform (http://huttenhower.sph.harvard.edu/galaxy) to perform the linear discriminant analysis effect size (LEFSe) and visualized the result using boxplots (R v3.6.1, ggplot2 package).

### Rarefaction curve analysis, diversity analyses, and enterotypes

To evaluate the gene richness in PV patients and healthy controls, a rarefaction curve was created. By randomly subsampling the cohort 30 times with replacement, we were able to calculate the gene richness from a given number of samples. We discovered that the gene richness progressively increased and leveled out as the sample size increased, demonstrating that the sample size was adequate. The process was implemented using the vegan package in R v3.6.1. α-Diversity was calculated based on the taxonomical abundance profile of each sample according to the Shannon index, while β-diversity was calculated using the Bray–Curtis distance. Furthermore, the genus abundance profile of the samples was subjected to permutational multivariate analysis of variance (PERMANOVA) (16) in order to compare the group of PV with healthy controls. We used Bray–Curtis distance and 9,999 permutations (R v3.6.1, vegan package). The enterotypes of each sample were analyzed by the Dirichlet multinomial mixture model-based method (17) using the DirichletMultinomial package in R v3.6.1.

### Co-occurrence network

Spearman’s rank correlation coefficient between differential species was calculated based on the species abundance profile. A network was then constructed by using the method implemented in Cytoscape v3.8.2. In the network, the edges indicate the correlation between two species, following the standard that Spearman’s rank correlation coefficient > 0.25 (blue line of the edge) or < -0.25 (red line of the edge).

### Random forest classifier

The random forest model with 10-fold cross-validation was established (R v3.6.1, randomForest package) using the differential species abundance profile. The average error curves from five trials of the 10-fold cross-validation were calculated. The cutoff was determined using the average curve’s smallest error plus the standard deviation at that point. We compiled a list of all species marker sets with errors lower than the cutoff value, and the set with the fewest species was chosen as the optimal set (18). The receiver operating characteristic curves were drawn using the pROC3 package in R v3.6.1.

### Functional analysis

For KEGG analysis, we first determined the KEGG Orthologs (KO) name of each gene by aligning the gene catalog to the eggNOG database using Diamond v2.0.5 (19) to expedite the protein sequence alignment procedure. By adding up the identical KOs, the relative abundance profile of KOs was obtained (a KO name contains multiple genes). Next, we downloaded the latest KEGG pathway list from the KEGG database (https://www.kegg.jp/keggbin/show_brite?ko00001.keg), enriched the KOs to the KEGG pathways, and screened the B- and C-level differential KEGG pathways between the PV and healthy control groups. The detailed screening method was as follows: 1) the KOs only expressed in less than 5 samples were removed, 2) different KOs were identified between the PV and healthy control groups (Benjamin–Hochberg *q*-value < 0.2, two-tail Wilcoxon sum-rank test), 3) the percentages of KO markers belonging to each KEGG category out of the total PV-enriched or control-enriched KO markers were calculated, and 4) Fisher’s exact test was used to calculate the significance level (20). Finally, KEGG with a *P* value < 0.05 was considered a different KEGG pathway between the PV and healthy control groups. Furthermore, we obtained significant MetaCyc pathways based on HUMAnN2 pipeline (34) and showed the contribution of specific microbial species to these pathways using the ggplot2 package in R v3.6.1.

### Detection of short-chain fatty acids

First, a 2 mL EP tube was filled with a 20 mg fecal sample that had been precisely weighed. The EP tube was filled with a milliliter of phosphoric acid solution (0.5% v/v) and a tiny steel ball. The mixture was three times ground for 10 seconds each, vortexed for 10 minutes, and ultrasonically heated for 5 minutes. The mixture was then centrifuged at 12000 rpm for 10 min at 4°C before being put into a 1.5 mL centrifuge tube with 0.1 mL of the supernatant. The centrifuge tube was filled with 0.5 mL of MTBE solution (including internal standard). The combination underwent 3 minutes of vortexing and 5 minutes of ultrasonication. The mixture was then centrifuged for 10 minutes at 4°C at a speed of 12000 rpm. GC-MS/MS analysis was performed using the supernatant that was obtained (21).

Then, an Agilent 7890B gas chromatograph coupled to a 7000D mass spectrometer with a DB-FFAP column (30 m length × 0.25 mm i.d. × 0.25 μm film thickness, J&W Scientific, USA) was employed for GC–MS/MS analysis of short-chain fatty acids (SCFAs). Helium was used as a carrier gas at a flow rate of 1.2 mL/min. The injection volume was 2 L, and the injection was performed in split mode. The oven temperature was kept at 90°C for one minute, then increased by 25°C every minute to 100°C, 20°C every minute to 150°C, held for 0.6 minutes, and then increased by 25°C every minute to 200°C, and held for 0.5 minutes after running for 3 minutes. All samples underwent numerous modalities of reaction monitoring analysis. The transfer line and injector inlet temperatures were 200 and 230 degrees Celsius, respectively (21, 22).

Finally, SCFA contents were detected by MetWare (http://www.metware.cn/) based on the Agilent 7890B-7000D GC–MS/MS platform.

### Statistical analysis

A significant difference in the study was determined if the *P* value was less than 0.05 (different KOs were identified based on the adjusted *P* value was less than 0.2 using the Benjamin-Hochberg method). The *P* value of α- and β-diversities were calculated by two-tail Wilcoxon sum-rank test and PERMANOVA, respectively. The two-tail Wilcoxon sum-rank test was also used to compare species and SCFAs in PV-healthy control groups and responder-non-responder groups, while paired t-test was performed to compare the pre- and post-treatment groups. We utilized the Kruskal-Wallis test for comparisons involving more than two groups. The difference in KEGG pathways between PV and healthy controls was determined using Fisher’s exact test. All correlation analyses were performed by using Spearman’s correlation analysis.

## Results

### Gut microbial dysbiosis in pemphigus vulgaris

We enrolled 60 PV patients and 19 healthy controls, who were comparable in age, sex, and body mass index (BMI) (**Table 1**). In addition, the patients and healthy controls shared their eating habits, lifestyles, and living environments to minimize the influence of non-PV-related factors. The characteristics of PV patients were summarized, including anti-Dsg 1/3 antibodies and affected skin/mucosa (**Table 1**). A total of 100 fecal samples were prospectively collected for metagenomic shotgun sequencing (treatment-naïve, n=60; post-treatment, n=21; healthy control, n = 19). The high-quality sequencing reads were aligned to a taxonomical database and then assembled *de novo*, and the identified genes were compiled into a nonredundant catalog of 4045593 genes for further functional analysis. Rarefaction analysis based on the 60 treatment-naïve PV patients and 19 healthy controls revealed that the gene richness approached saturation in each group, implying that the sample size of each group was adequate (**Figure S1A**).

**Table 1:** Demographic and clinical information of patients with PV and healthy controls.

	PV (n=60)	Healthy controls (n=19)	*P* value
Sex			
Female, n (%)	33 (55.00)	9 (47.37)	0.75
Male, n (%)	27 (45.00)	10 (52.63)	
Age, years, mean (S.D.)	47.38 (12.82)	41.00 (14.81)	0.10
BMI, kg/m^2^, mean (S.D.)	22.52 (2.81)	23.44 (3.28)	0.28
Mocosa affected			
Yes, n (%)	53 (88.33)	\	\
No, n (%)	7 (11.67)	\	
Skin affected			
Yes, n (%)	57 (95.00)	\	\
No, n (%)	3 (5.00)	\	
PDAI			
Before treatment, mean (S.D.)	20.09 (14.43)	\	\
After treatment, mean (S.D.)	8.65 (8.67)	\	\
Anti-Dsg1 antibody, μ/mL, mean (S.D.)	108.85 (66.56)	\	\
Anti-Dsg3 antibody, μ/mL, mean (S.D.)	123.8 (63.72)	\	\

PV, pemphigus vulgaris; BMI, body mass index; PDAI, pemphigus disease area index; Dsg, desmoglein. The symbol "\" means healthy controls did not have these PV related features.

**Figure 1:**
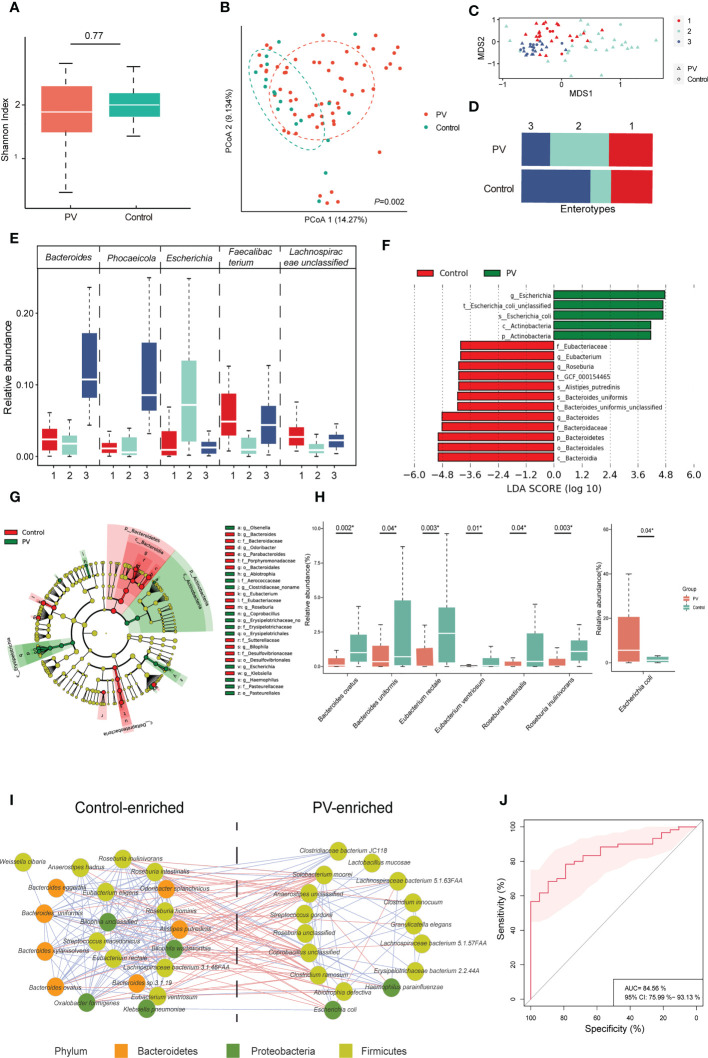
Diversities, enterotypes and differential microbial species between PV and healthy controls. **(A, B)** The α-diversity and β-diversity of the gut microbiome in the PV and healthy control groups; **(C)** The distribution of the three enterotypes is shown in descending order using non-metric multidimensional scaling; Red, green and blue represent enterotype1, enterotype2, and enterotype3 respectively, while the triangle represents patients with PV and the circle represents healthy controls; **(D)** Proportion of enterotypes distribution in PV and healthy controls; **(E)** The dominant genus of each enterotype; **(F, G)** Results of differential bacteria between PV and healthy controls by LEFse analysis; **(H)** Main differential species between the healthy control and PV groups (* represents statistical significance with the *P* value <0.05 using Wilcoxon sum-rank test); **(I)** The interaction of differential species between patients with PV and healthy controls, with the red line representing negative correlations and the blue line representing positive correlations; only species with absolute values of correlation coefficients greater than 0.25 are shown; **(J)** Species-based identification of pemphigus vulgaris; ROC curve for the set with an AUC of 84.56% and the 95% CI of 75.99% to 93.13%. PV, pemphigus vulgaris; PCoA, principal coordinates analysis; LEFse, LDA effect size analysis; LDA, linear discriminant analysis; ROC, receiver operating characteristic curve; AUC, the area under the receiver operating curve; CI, confidence interval.

Next, we investigated the microbial community differences between PV and controls in terms of microbiome diversity. Differences were not observed for α-diversity (*P=*0.77, Wilcoxon sum-rank test, **Figure 1A**), whereas β-diversity reached statistical significance (*P*=0.002, PERMANOVA, **Figure 1B**), suggesting that the compositions of the gut microbiome rather than the species richness of microbial communities were altered in PV patients compared to healthy family members. We also performed enterotype analysis to assess the global alterations of the gut microbiome, dividing these participants into three different enterotype groups: (i.e., E1, E2, and E3) (**Figures 1C**, **S1B**). Despite all three enterotypes being present in both PV patients and healthy controls, E2 and E3 were significantly enriched in PV patients and controls, respectively (*P* = 0.02, Chi-squared test) (**Figure 1D**). Specifically, these three enterotypes were attributed to the relative abundance of different microbial species, such as *Faecalibacterium* (predominant in E1), *Escherichia* (predominant in E2), and *Bacteroides* (predominant in E3) (**Figure 1E**), confirming that the composition of the gut microbiome in PV deviated from that of healthy controls.

To further elucidate the specific bacteria that differed between PV patients and healthy controls, we performed LEfSe analysis and identified PV- and control-enriched bacteria at each level (**Figures 1F, G**; **Table S2**). For instance, at the phylum level, *Actinobacteria* and *Bacteroidetes* were more abundant in the PV patients and healthy controls, respectively (**Figures S1C, E**; **Table S2**). At the genus level, *Bacteroides* was enriched in the healthy control whereas *Escherichia* was enriched in the PV patients, which is consistent with a previous report on the gut microbiome of PV (13) (**Figures S1D, F**; **Table S2**). Finally, at the species level, we found a significant decrease in the relative abundance of probiotics in PV patients compared to healthy controls, including *Bacteroides ovatus*, *Bacteroides uniformis*, and some SCFA-producing bacteria, such as *Eubacterium rectale*, *Eubacterium ventriosum*, *Roseburia intestinalis*, and *Roseburia inulinivorans* (**Figure 1H**; **Table S2**), whereas the number of enriched bacteria in PV patients were small, mainly including *Escherichia coli* (**Figure 1H** and **Table S2**). Moreover, the control/PV-enriched microbial species exhibited positive internal correlations with each other, whereas they tended to have negative correlations between the two groups (**Figure 1I**), suggesting an antagonistic or mutually exclusive relationship. Finally, we constructed a random forest classifier to demonstrate the diagnostic potential of the gut microbiome for PV. Five repeats of 10-fold cross-validation on the training set led to the optimal selection of 28 species markers with an area under the receiver operating curve (AUC) of 84.56% (**Figure 1J**, **S1G**).

Noteworthy, we obtained consistent results in the subgroup analysis specifically with PV and their matched family member controls, as shown by the fact that the gut microbiome between the two groups revealed substantial variations in β-diversity (*P*=0.01, PERMANOVA, **Figure S2A**) but not in α-diversity (*P*=0.78, Wilcoxon sum-rank test, **Figure S2A**). Moreover, the different species between the two groups were consistent with those indicated above between the PV and healthy control groups (**Figures S2B, C**), including *Escherichia coli*, *Bacteroides ovatus*, *Eubacterium rectale, Eubacterium ventriosum*, *Roseburia intestinalis*, and *Roseburia inulinivorans*. This consistent finding demonstrates the accuracy of the analysis results and eliminates the possible association of these specific bacteria with characteristics unrelated to disease, such as sample size, eating habits, lifestyles, and living environments.

### Correlation of gut microbiome with clinical features of PV

**Figure 2:**
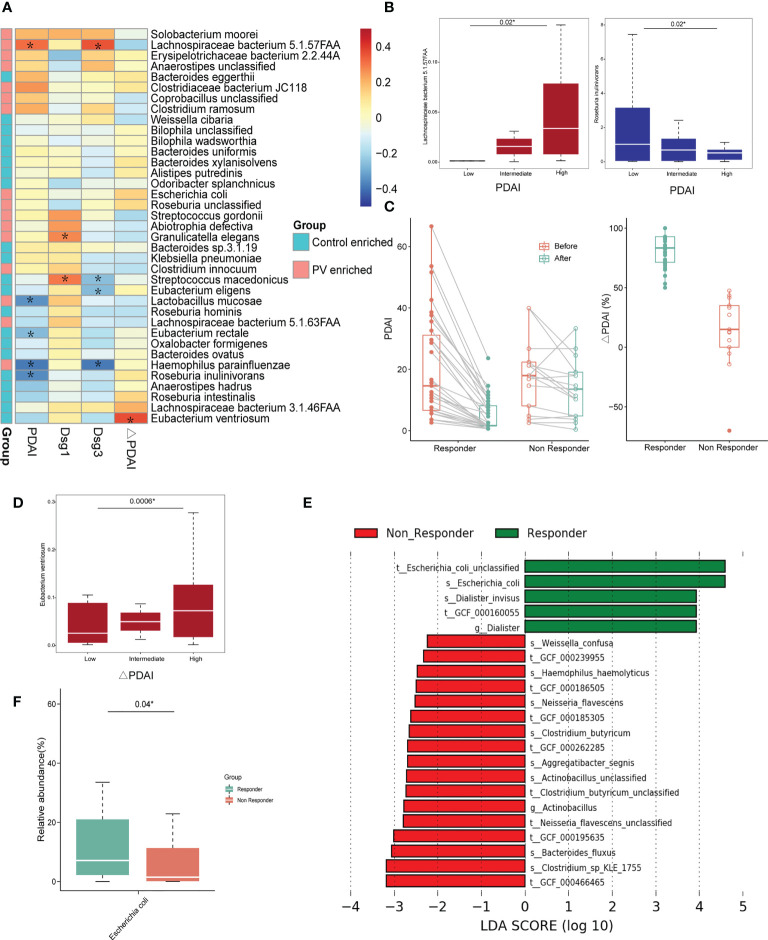
The correlation between differential microbial species and clinical indexes. **(A)** Heatmap of correlation coefficients between differential microbial species and PDAI, anti-Dsg1 antibody titers, anti-Dsg3 antibody titers, and ΔPDAI; The *means the *P* value is less than 0.05 using Spearman’s correlation analysis; **(B)** Positive correlation between PV-enriched gut microbiome and PDAI scores and negative correlation between the healthy control-enriched gut microbiome and PDAI scores (*represents statistical significance with the *P* value <0.05 using Kruskal-Wallis test); **(C)** The graph on the left shows the change in PDAI scores before and after treatments in the responder and non-responder groups; The graph on the right shows the ΔPDAI for the responder and non-responder groups; **(D)** The positive correlation between the relative abundance of *Eubacterium ventriosum* and ΔPDAI (*represents statistical significance with the *P* value <0.05 using Kruskal-Wallis test); **(E–G)** Results of differential bacteria between the responder and non-responder groups by LEFse analysis; **(F)** Boxplots of the relative abundance of *Escherichia coli* in the responder and non-responder groups (*represents statistical significance with the *P* value <0.05 using Wilcoxon sum-rank test). PV, pemphigus vulgaris; PDAI, pemphigus disease area index; Dsg: desmoglein; ΔPDAI: PDAI improvement rates; LEFse, LDA effect size analysis; LDA, linear discriminant analysis.

Since disease severity in PV patients positively correlates with PDAI scores and anti-Dsg antibody titers, we estimated the correlation of differential species with clinical indexes of PV (**Figure 2A**). Only a few of the varied microbial species exhibited significant association, including a positive correlation of *Lachnospiraceae bacterium 5.1.57FAA* abundance with anti-Dsg3 antibodies (R = 0.35, *P* = 0.005, Spearman) and PDAI scores (R = 0.32, *P*=0.02, Spearman), and negative correlation of PDAI scores with the abundance of *Eubacterium rectale* (R = -0.26, *P* = 0.04, Spearman) and *Roseburia inulinivorans* (R = -0.36, *P* = 0.01, Spearman) (**Figure 2A**), which also exhibited the same trend with categorical division (**Figure 2B**). Therefore, these findings demonstrated the possible link between the gut microbiome and PV severity.

Given that the response to conventional glucocorticoid-based treatment can be reflected by the ΔPDAI (**Figure 2C**), we estimated the correlation between ΔPDAI and each microbiota component to evaluate the potential prognostic value of the microbial species with response outcomes. In the linear model, *Eubacterium ventriosum* exhibited the strongest positive correlation with the ΔPDAI (R=0.36, *P*=0.01, Spearman) (**Figure 2A**), consistent with the categorical division (**Figure 2D**). With LEfSe analysis in a categorical model, *Escherichia coli* was found to be the predominant microbial species in the responder group, compared to the non-responder group (**Figures 2E, F**). Paradoxically, *Escherichia coli* is a pathogenic bacterium that is enriched in PV patients compared to healthy controls and showed higher abundance in responders than non-responders, suggesting its possible bi-directional role in PV onset and treatment response.

### Glucocorticoid partially altered the gut microbiome in PV patients

**Figure 3:**
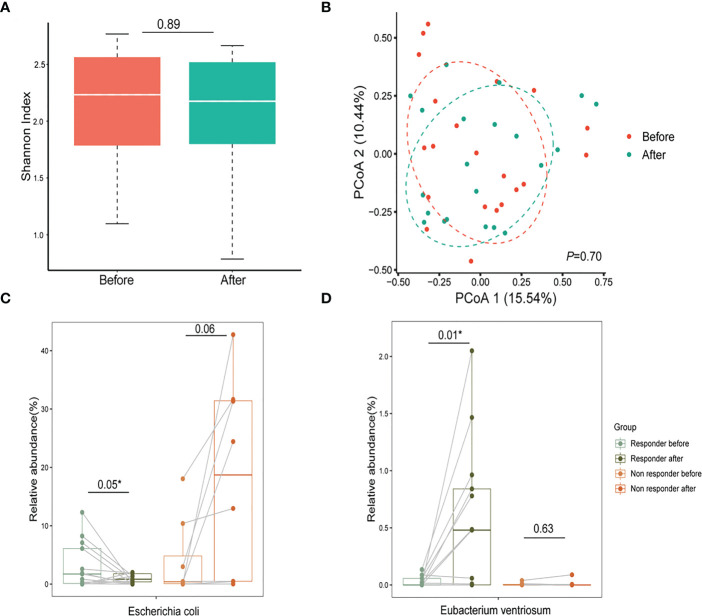
Glucocorticoids aid in reestablishing a healthy gut microbiome in patients with PV. **(A, B)** The α-diversity and β-diversity of the gut microbiome in the pre-treatment and post-treatment groups; **(C)** After one month of glucocorticoid therapy, *Escherichia coli* decreased in relative abundance in the responder group and increased in the non-responder group (* represents statistical significance with the *P* value <0.05 using paired t-test); **(D)** The relative abundance of probiotics (*Eubacterium ventriosum*) reduced in the PV group increased after treatment with glucocorticoids (*represents statistical significance with the *P* value <0.05 using paired t-test). PV, pemphigus vulgaris; PCoA, principal coordinates analysis.

To examine the effect of glucocorticoid treatment on the gut microbiome in PV patients, we compared the gut microbiome of 21 pairs of matched fecal samples collected before and after one month of treatment, particularly focusing on the relative abundance of PV-related bacteria. Both α-diversity and β-diversity did not differ significantly between pre-and post-treatment samples (**Figures 3A, B**), suggesting the weak overall interaction of glucocorticoid treatment and microbiome. However, some PV-related bacteria were altered after treatment, particularly in responders. For instance, the relative abundance of *Escherichia coli* decreased in responders after treatment (*P*=0.05, paired t-test, **Figure 3C**), whereas the probiotics abundance (e.g., *Eubacterium ventriosum*) was specifically elevated in responders (*P*=0.01, paired t-test, **Figure 3D**), implying that glucocorticoid treatment might help inhibit *Escherichia coli* and reestablish a healthy gut microbiome in responders. However, such an impact might not be achievable in non-responders because they were resistant to glucocorticoid medication. The precise mechanisms underlying resistance to glucocorticoid therapy in these patients remain to be further explored in the future.

### Functional changes in the gut microbiome

**Figure 4:**
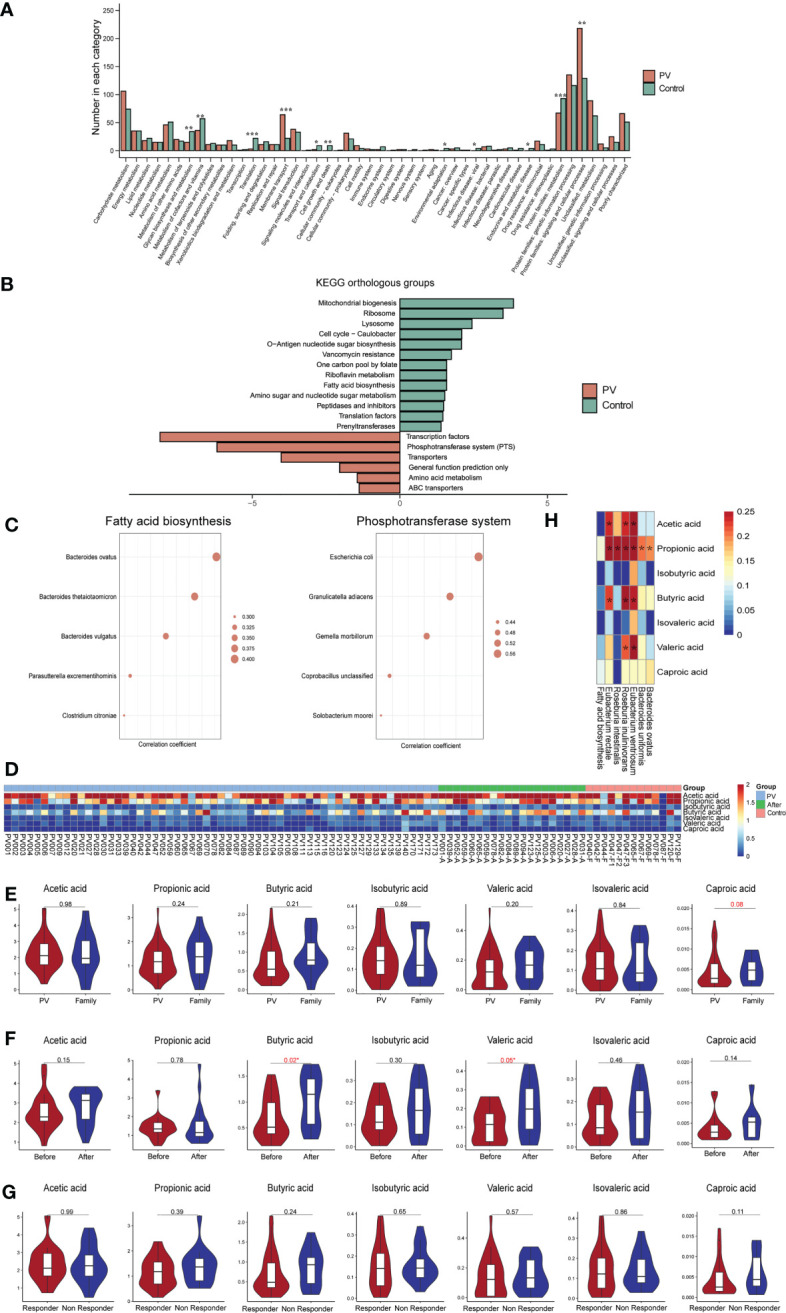
Functional changes in the gut microbiome. **(A)** Level B differential KEGG pathways, horizontal coordinates represent pathway names, vertical coordinates represent the number of differential KOs involved in each pathway (*represents a *P* value less than 0.05, **represents a *P* value less than 0.005, ***represents a *P* value less than 0.0005); **(B)** C-level difference KEGG pathway between PV patients and healthy controls, horizontal coordinate represents the *P* value of -log10; **(C)** The top five microbial species with the strongest association with fatty acid biosynthesis and phosphotransferase system pathways; **(D)** Heatmap of short fatty acids contents in each sample; **(E–G)** SCFA levels in the **(E)** PV and healthy control groups, **(F)** pre-treatment and post-treatment groups, and **(G)** responder and non-responder groups; **(H)** Heatmap of correlation coefficients between healthy control-enriched probiotics and SCFA levels; The *means the *P* value is less than 0.05 using Spearman’s correlation analysis. PV, pemphigus vulgaris; KEGG, Kyoto encyclopedia of genes and genomes; KO, KEGG Orthology; SCFA, short chain fatty acid.

We conducted KEGG analysis and found that PV-enriched KO markers were typically involved in the KEGG B-level categories of membrane transport and protein families involving signaling/cellular processes, whereas healthy control-enriched KO markers were frequently involved in translation and protein families involved in metabolism (**Figure 4A**). At the level of KEGG class C, 13 and 6 pathways were enriched in the healthy family members and PV patients, respectively (**Figure 4B**). Intriguingly, the phosphotransferase system (PTS) pathway, which can regulate the virulence of pathogenic bacteria, was highly presented in PV patients, and had the strongest correlation with *Escherichia coli* (R=0.59, *P*=1.58e-10, Spearman, **Figure 4C**), whereas fatty acid biosynthesis pathway was enriched in healthy controls and had the strongest correlation with *Bacteroides ovatus* (R=0.41, *P*= 2.67e-05, Spearman, **Figure 4C**), suggesting the two functional pathways attributed to *Escherichia coli/Bacteroidetes* dominant effects. Furthermore, among MetaCyc pathways, we also found *Bacteroidetes* mainly contributed to the fatty acid biosynthesis pathway, and apart from PTS*, Escherichia coli* had predominance in enterobactin biosynthesis pathways (**Figures S3A, B**). We further performed SCFA-targeted metabolomics analysis to investigate the relationship between SCFA components and PV onset and treatment outcomes. With available leftover fecal samples from metagenomic sequencing (treatment-naïve, n = 59; post-treatment, n = 20, healthy relatives, n = 13) (**Figure 4D**), healthy controls had higher or comparable SCFA levels compared to PV patients (**Figure 4E**). Particularly, butyric acid (*P* = 0.02, paired t-test) and valeric acid (*P* = 0.05, paired t-test) rebounded significantly after one month of glucocorticoid treatment in PV patients (**Figure 4F**). However, there were no significant differences in any SCFA levels between the responder and non-responder of PV patients (**Figure 4G**). It is worth mentioning that healthy control-enriched probiotics had significant correlations with SCFA levels (**Figure 4H**), which suggests that the vital function of these probiotics might to produce SCFA.

## Discussion

In this study, we explored the PV-related gut microbiome alteration that are associated with both onset and treatment response. Moreover, the decreased SCFA level revealed by targeted metabolomics is strongly correlated with decreased SCFA-producing probiotics in fecal samples from PV patients. To the best of our knowledge, this is the first comprehensive investigation of the gut microbiome and microbiome-derived metabolites of PV, which provide a basis for the possible noninvasive diagnostic approach and interventions for the risk reduction and treatment of PV.

Among all differential gut microbial species, *Escherichia coli* tends to play the most significant role in PV, as it was not only abundant in PV patients and decreased after conventional treatment, but also enriched in responders compared to non-responders, suggesting that PV onset in some patients may be attributed to *Escherichia coli* and sensitive to glucocorticoid treatment. *Escherichia coli* is a member of the *Proteobacteria* phylum, *gamma-Proteobacteria* class, and *Escherichia* genus, the level of which has been consistently linked to PV onset in a previous study (13). As a gram-negative bacterium, the outer membrane surface of *Escherichia coli* is coated with lipopolysaccharide (LPS), a complex glycolipid that is a major bacterial virulence factor. LPS can induce a robust proinflammatory response and the secretion of proinflammatory cytokines, which may lead to the disruption of the intestinal barrier. The impaired intestinal barrier allows the passage of toxins, antigens, and bacteria to pass into the lumen and enter the bloodstream, triggering the onset and progression of autoimmune disease (35–37). Moreover, we observed that *Escherichia coli* played a dominant contribution role in the enrichment of PTS pathway in PV patients. The PTS pathway has been proved to modulate the expression of virulence genes in pathogenic bacteria, such as *Vibrio cholerae* (38) and *Bacillus anthracis* (39). Therefore, we speculated that PTS may also increase the virulence gene expression of *Escherichia coli*, which increases its pathogenicity and then exacerbates the disruption of the intestinal barrier in PV patients. Given that glucocorticoid (e.g., prednisolone) does not exhibit direct *in vitro* antibacterial activity against *Escherichia coli* (23), the decrease of gut *Escherichia coli* after glucocorticoid treatment are more likely to be influenced by its regulatory role in the immune system, but the mechanism remains to be elucidated.

On the other hand, we observed a consistent decrease in the relative abundance of multiple probiotics, including *Bacteroides* (e.g., *Bacteroides uniformis*, *Bacteroides ovatus*) and several SCFA-producing bacteria (e.g., *Eubacterium ventriosum*), which might be due to the inhibitory effect of *Escherichia coli*. Our results demonstrated that these probiotics had a mutually exclusive relationship with *Escherichia coli*, which could be explained by the speculation that *Escherichia coli*-dominated enterobactin biosynthesis pathway was enriched in PV patients and the production of which could promote the own growth of *Escherichia coli* by ingesting iron while inhibiting the growth of other bacteria (40). Previous studies have shown that many species of *Bacteroides* can alleviate LPS-induced inflammation and improve gut barrier function (37, 41, 42). Therefore, a lack of *Bacteroides* can exacerbate the inflammation induced by LPS in *Escherichia coli*, resulting in a worsening of intestinal barrier disruption and an increase in intestinal inflammation levels. Meanwhile, we noted a decline of SCFAs in PV patients, which was significantly correlated with the decreased abundance of healthy control-enriched probiotics. The important roles of SCFA have been established in modulating immune/inflammatory responses and intestinal barrier function (29). Particularly, butyric acid can act as an anti-inflammatory agent and maintain the balance of tolerance to commensals and immunity to pathogenic bacteria in the intestinal immune system (43). The decline of SCFA levels in PV patients could further impair intestinal barrier function and promote the disease progression, while glucocorticoids can partially restore the SCFA-producing bacteria and the SCFA level (e.g., butyric acid) in responders. Our results suggested that glucocorticoids may effectively improve microbial and metabolite dysbiosis and strengthen the intestinal barrier, thereby aiding in the restoration of intestinal homeostasis in PV patients. Also, it is worth determining whether supplementing SCFAs can render refractory PV patients responsive to conventional treatment in the future.

This is the first study to combine the use of metagenomic and metabolomic technologies to investigate the relationship between the gut microbiome/metabolites and onset/treatment response of PV. We concluded a potential mechanism for PV onset: increased pathogenic *Escherichia coli* secreting toxins and enterobactin could disrupt the intestinal barrier and inhibit numerous probiotics. As a result, the gut microbiome is imbalanced and SCFA levels significantly decrease in PV patients, which further impairs the intestinal barrier function. We speculated that pathogenic bacteria/toxins might then pass through the damaged intestinal barrier and trigger the initiation and development of PV through antigen mimicry, inducing immune responses, etc. However, our study still has several limitations. First, since the research is cross-sectional, we were only able to determine the correlation, not the causality of the gut microbiome. Second, as a single-center study with relatively small sample sizes, our findings need further support and verifications by independent studies. Thirdly, due to the lack of a control group for other autoimmune diseases in this study, the differential species identified in this study may be shared by other autoimmune diseases but not unique to PV. Finally, the mechanisms by which *Escherichia coli* and toxins subsequently trigger the immune process of PV and the implications of SCFA supplementation for PV treatment will need to be further explored in subsequent studies.

In conclusion, our comprehensive investigation of the gut microbiome in PV patients suggests an association of PV onset with the enrichment of pathogenic bacteria and a lack of probiotics (e.g., SCFA-producing bacteria), which can be partially restored towards healthy individuals after glucocorticoid treatment. Future *in vivo* and *in vitro* experiments are required to elucidate the causal relationship between the gut microbiome and PV, as well as possible mechanisms.

## Data availability statement

The data presented in the study are deposited in the database (https://bigd.big.ac.cn/gsa-human/browse/), accession number HRA004017.

## Ethics statement

The studies involving human participants were reviewed and approved by Ethics committee of the West China Hospital, Sichuan University, approval no. 2017 (241)). The patients/participants provided their written informed consent to participate in this study.

## Author contributions

Conception and design: YW, XX, WL, and HX; Administrative support: WL and HX; Collection of study materials or patient samples: YW, XZ, TZ, YZ, and WL. Collection and assembly of data: YW, XX, WL, and YS; Data analysis and interpretation: YW, XX, QD, and WZ; Funding acquisition: WZ, WL, and HX; All authors contributed to the article and approved the submitted version.

## Supplementary material

Supplementary Figure 1 Diversities, enterotypes, and differential species between the PV and healthy control groups. **(A)** The rarefaction curve showed that the gene richness approached saturation in each group with red representing PV and green representing healthy control; **(B)** Three enterotypes were clustered using the DMM model; **(C, E)** Differential phylum between the healthy control and PV groups; **(D, F)** Differential genus between the healthy control and PV groups; **(G)** Contribution of each species to the random forest model in the optimal set. PV, pemphigus vulgaris; DMM, Dirichlet Multinomial Mixture Model.

Supplementary Figure 2 Subgroup analysis specifically with PV and their matched family member controls. **(A)** The α-diversity (left) and β-diversity (right) of the gut microbiome in the PV patients and their matched family members; **(B)** Results of differential bacteria between PV and family members by LEFse analysis; **(C)** Main differential species between PV patients and their family members (* represents statistical significance with the P value <0.05 using Wilcoxon sum-rank test); **(D)** C-level difference KEGG pathway between PV patients and their matched family members, horizontal coordinate represents the P value of -log10. PV, pemphigus vulgaris; PCoA, principal coordinates analysis; LEFse, LDA effect size analysis; LDA, linear discriminant analysis; KEGG, Kyoto encyclopedia of genes and genomes.

Supplementary Figure 3 Different MetaCyc pathways between PV patients and healthy controls. **(A)** Contribution of the microbial genus to the fatty acid biosynthesis pathway; **(B)** Contribution of the microbial species to the enterobactin biosynthesis pathway.

Supplementary Table 1 Clinical information for all samples.

Supplementary Table 2 Microbial phylum, genus, and species associated with pemphigus vulgaris.

## References

[1] Schmidt E Kasperkiewicz M Joly P. Pemphigus. Lancet (2019) 394(10201):882–94. doi: 10.1016/s0140-6736(19)31778-7 31498102

[2] Kasperkiewicz M Ellebrecht CT Takahashi H Yamagami J Zillikens D Payne AS. Pemphigus. Nat Rev Dis Primers (2017) 3:17026. doi: 10.1038/nrdp.2017.26 28492232 PMC5901732

[3] Stanley JR Amagai M. Pemphigus, bullous impetigo, and the staphylococcal scalded-skin syndrome. N Engl J Med (2006) 355(17):1800–10. doi: 10.1056/NEJMra061111 17065642

[4] Gao J Zhu C Zhang Y Sheng Y Yang F Wang W. Association study and fine-mapping major histocompatibility complex analysis of pemphigus vulgaris in a han chinese population. J Invest Dermatol (2018) 138(11):2307–14. doi: 10.1016/j.jid.2018.05.011 29857070

[5] Zhang SY Zhou XY Zhou XL Zhang Y Deng Y Liao F. Subtype-specific inherited predisposition to pemphigus in the Chinese population. Br J Dermatol (2019) 180(4):828–35. doi: 10.1111/bjd.17191 30230522

[6] Sinha AA. The genetics of pemphigus. Dermatol Clin (2011) 29(3):381–91. doi: 10.1016/j.det.2011.03.020 21605803

[7] Khan MF Wang H. Environmental exposures and autoimmune diseases: Contribution of gut microbiome. Front Immunol (2019) 10:3094. doi: 10.3389/fimmu.2019.03094 31998327 PMC6970196

[8] Zhang X Chen BD Zhao LD Li H. The gut microbiota: Emerging evidence in autoimmune diseases. Trends Mol Med (2020) 26(9):862–73. doi: 10.1016/j.molmed.2020.04.001 32402849

[9] Alpizar-Rodriguez D Lesker TR Gronow A Gilbert B Raemy E Lamacchia C. Prevotella copri in individuals at risk for rheumatoid arthritis. Ann Rheum Dis (2019) 78(5):590–3. doi: 10.1136/annrheumdis-2018-214514 30760471

[10] Azzouz D Omarbekova A Heguy A Schwudke D Gisch N Rovin BH. Lupus nephritis is linked to disease-activity associated expansions and immunity to a gut commensal. Ann Rheum Dis (2019) 78(7):947–56. doi: 10.1136/annrheumdis-2018-214856 PMC6585303 30782585

[11] Chen BD Jia XM Xu JY Zhao LD Ji JY Wu BX. An autoimmunogenic and proinflammatory profile defined by the gut microbiota of patients with untreated systemic lupus erythematosus. Arthritis Rheumatol (2021) 73(2):232–43. doi: 10.1002/art.41511 33124780

[12] Zhou C Zhao H Xiao XY Chen BD Guo RJ Wang Q. Metagenomic profiling of the pro-inflammatory gut microbiota in ankylosing spondylitis. J Autoimmun (2020) 107:102360. doi: 10.1016/j.jaut.2019.102360 31806420

[13] Huang S Mao J Zhou L Xiong X Deng Y. The imbalance of gut microbiota and its correlation with plasma inflammatory cytokines in pemphigus vulgaris patients. Scand J Immunol (2019) 90(3):e12799. doi: 10.1111/sji.12799 31211854 PMC9286422

[14] Han D Gao P Li R Tan P Xie J Zhang R. Multicenter assessment of microbial community profiling using 16s rrna gene sequencing and shotgun metagenomic sequencing. J Adv Res (2020) 26:111–21. doi: 10.1016/j.jare.2020.07.010 PMC7584675 33133687

[15] Durazzi F Sala C Castellani G Manfreda G Remondini D De Cesare A. Comparison between 16s rrna and shotgun sequencing data for the taxonomic characterization of the gut microbiota. Sci Rep (2021) 11(1):3030. doi: 10.1038/s41598-021-82726-y 33542369 PMC7862389

[16] Mallick H Ma S Franzosa EA Vatanen T Morgan XC Huttenhower C. Experimental design and quantitative analysis of microbial community multiomics. Genome Biol (2017) 18(1):228. doi: 10.1186/s13059-017-1359-z 29187204 PMC5708111

[17] Weersma RK Zhernakova A Fu J. Interaction between drugs and the gut microbiome. Gut (2020) 69(8):1510–9. doi: 10.1136/gutjnl-2019-320204 PMC7398478 32409589

[18] Zhou B Xia X Wang P Chen S Yu C Huang R. Induction and amelioration of methotrexate-induced gastrointestinal toxicity are related to immune response and gut microbiota. EBioMedicine (2018) 33:122–33. doi: 10.1016/j.ebiom.2018.06.029 PMC6085585 30049384

[19] Yu C Zhou B Xia X Chen S Deng Y Wang Y. Prevotella copri is associated with carboplatin-induced gut toxicity. Cell Death Dis (2019) 10(10):714. doi: 10.1038/s41419-019-1963-9 31558709 PMC6763498

[20] Gopalakrishnan V Spencer CN Nezi L Reuben A Andrews MC Karpinets TV. Gut microbiome modulates response to anti-Pd-1 immunotherapy in melanoma patients. Science (2018) 359(6371):97–103. doi: 10.1126/science.aan4236 29097493 PMC5827966

[21] Dubin K Callahan MK Ren B Khanin R Viale A Ling L. Intestinal microbiome analyses identify melanoma patients at risk for checkpoint-Blockade-Induced colitis. Nat Commun (2016) 7:10391. doi: 10.1038/ncomms10391 26837003 PMC4740747

[22] Yan X Zhang S Deng Y Wang P Hou Q Xu H. Prognostic factors for checkpoint inhibitor based immunotherapy: An update with new evidences. Front Pharmacol (2018) 9:1050. doi: 10.3389/fphar.2018.01050 30294272 PMC6159743

[23] Maier L Pruteanu M Kuhn M Zeller G Telzerow A Anderson EE. Extensive impact of non-antibiotic drugs on human gut bacteria. Nature (2018) 555(7698):623–8. doi: 10.1038/nature25979 PMC6108420 29555994

[24] Dai Q Xia X He C Huang Y Chen Y Wu Y. Association of anti-Tnf-α treatment with gut microbiota of patients with ankylosing spondylitis. Pharmacogenet Genomics (2022) 32(7):247–56. doi: 10.1097/fpc.0000000000000468 PMC9351697 35852868

[25] Yin J Sternes PR Wang M Song J Morrison M Li T. Shotgun metagenomics reveals an enrichment of potentially cross-reactive bacterial epitopes in ankylosing spondylitis patients, as well as the effects of tnfi therapy upon microbiome composition. Ann Rheum Dis (2020) 79(1):132–40. doi: 10.1136/annrheumdis-2019-215763 31662318

[26] Chen Z Zheng X Wu X Wu J Li X Wei Q. Adalimumab therapy restores the gut microbiota in patients with ankylosing spondylitis. Front Immunol (2021) 12:700570. doi: 10.3389/fimmu.2021.700570 34539629 PMC8441001

[27] Holmes E Li JV Athanasiou T Ashrafian H Nicholson JK. Understanding the role of gut microbiome-host metabolic signal disruption in health and disease. Trends Microbiol (2011) 19(7):349–59. doi: 10.1016/j.tim.2011.05.006 21684749

[28] Chang AE Golob JL Schmidt TM Peltier DC Lao CD Tewari M. Targeting the gut microbiome to mitigate immunotherapy-induced colitis in cancer. Trends Cancer (2021) 7(7):583–93. doi: 10.1016/j.trecan.2021.02.005 33741313

[29] Kim CH. Control of lymphocyte functions by gut microbiota-derived short-chain fatty acids. Cell Mol Immunol (2021) 18(5):1161–71. doi: 10.1038/s41423-020-00625-0 PMC8093302 33850311

[30] Lu Y Yuan X Wang M He Z Li H Wang J. Gut microbiota influence immunotherapy responses: Mechanisms and therapeutic strategies. J Hematol Oncol (2022) 15(1):47. doi: 10.1186/s13045-022-01273-9 35488243 PMC9052532

[31] Agus A Clément K Sokol H. Gut microbiota-derived metabolites as central regulators in metabolic disorders. Gut (2021) 70(6):1174–82. doi: 10.1136/gutjnl-2020-323071 PMC8108286 33272977

[32] Lavelle A Sokol H. Gut microbiota-derived metabolites as key actors in inflammatory bowel disease. Nat Rev Gastroenterol Hepatol (2020) 17(4):223–37. doi: 10.1038/s41575-019-0258-z 32076145

[33] Harman KE Brown D Exton LS Groves RW Hampton PJ Mohd Mustapa MF. British Association of dermatologists' guidelines for the management of pemphigus vulgaris 2017. Br J Dermatol (2017) 177(5):1170–201. doi: 10.1111/bjd.15930 29192996

[34] Franzosa EA McIver LJ Rahnavard G Thompson LR Schirmer M Weingart G. Species-level functional profiling of metagenomes and metatranscriptomes. Nat Methods (2018) 15(11):962–8. doi: 10.1038/s41592-018-0176-y PMC6235447 30377376

[35] Mu Q Kirby J Reilly CM Luo XM. Leaky gut as a danger signal for autoimmune diseases. Front Immunol (2017) 8:598. doi: 10.3389/fimmu.2017.00598 28588585 PMC5440529

[36] Petta I Fraussen J Somers V Kleinewietfeld M. Interrelation of diet, gut microbiome, and autoantibody production. Front Immunol (2018) 9:439. doi: 10.3389/fimmu.2018.00439 29559977 PMC5845559

[37] Hiippala K Jouhten H Ronkainen A Hartikainen A Kainulainen V Jalanka J. The potential of gut commensals in reinforcing intestinal barrier function and alleviating inflammation. Nutrients (2018) 10(8):988. doi: 10.3390/nu10080988 30060606 PMC6116138

[38] Wang Q Millet YA Chao MC Sasabe J Davis BM Waldor MK. A genome-wide screen reveals that the vibrio cholerae phosphoenolpyruvate phosphotransferase system modulates virulence gene expression. Infect Immun (2015) 83(9):3381–95. doi: 10.1128/iai.00411-15 PMC4534656 26056384

[39] Stülke J. Regulation of virulence in bacillus anthracis: The phosphotransferase system transmits the signals. Mol Microbiol (2007) 63(3):626–8. doi: 10.1111/j.1365-2958.2006.05556.x 17302796

[40] Raymond KN Dertz EA Kim SS. Enterobactin: An archetype for microbial iron transport. PNAS (2003) 100(7):5. doi: 10.1073/pnas.0630018100 PMC152965 12655062

[41] Tan H Zhao J Zhang H Zhai Q Chen W. Novel strains of bacteroides fragilis and bacteroides ovatus alleviate the lps-induced inflammation in mice. Appl Microbiol Biotechnol (2019) 103(5):2353–65. doi: 10.1007/s00253-019-09617-1 30666361

[42] Hiippala K Kainulainen V Suutarinen M Heini T Bowers JR Jasso-Selles D. Isolation of anti-inflammatory and epithelium reinforcing bacteroides and parabacteroides spp. from a healthy fecal donor. Nutrients (2020) 12(4):935. doi: 10.3390/nu12040935 32230951 PMC7230855

[43] Fu X Liu Z Zhu C Mou H Kong Q. Nondigestible carbohydrates, butyrate, and butyrate-producing bacteria. Crit Rev Food Sci Nutr (2019) 59(sup1):S130–s52. doi: 10.1080/10408398.2018.1542587 30580556

