# Supplementary figures and images for: Association of gut microbiome and metabolites with onset and treatment response of patients with pemphigus vulgaris
Source: Front Immunol. 2023 Apr 14;14:1114586. doi: 10.3389/fimmu.2023.1114586 (PMC10140300; doi:10.3389/fimmu.2023.1114586)

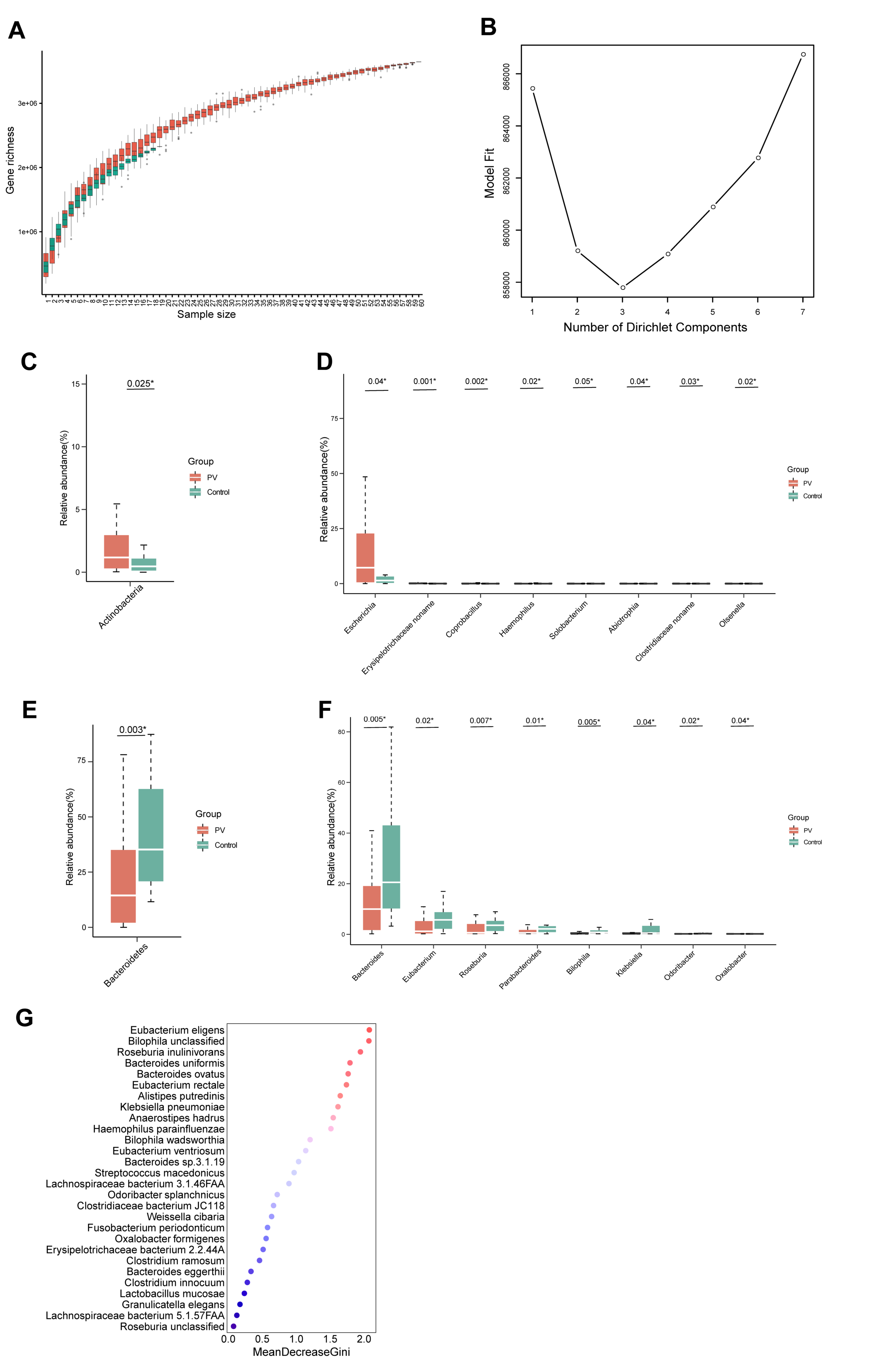

Supplement: Supplementary Figure 1 — Diversities, enterotypes, and differential species between the PV and healthy control groups. (A) The rarefaction curve showed that the gene richness approached saturation in each group with red representing PV and green representing healthy control; (B) Three enterotypes were clustered using the DMM model; (C, E) Differential phylum between the healthy control and PV groups; (D, F) Differential genus between the healthy control and PV groups; (G) Contribution of each species to the random forest model in the optimal set. PV, pemphigus vulgaris; DMM, Dirichlet Multinomial Mixture Model. [file Image_1.tif]

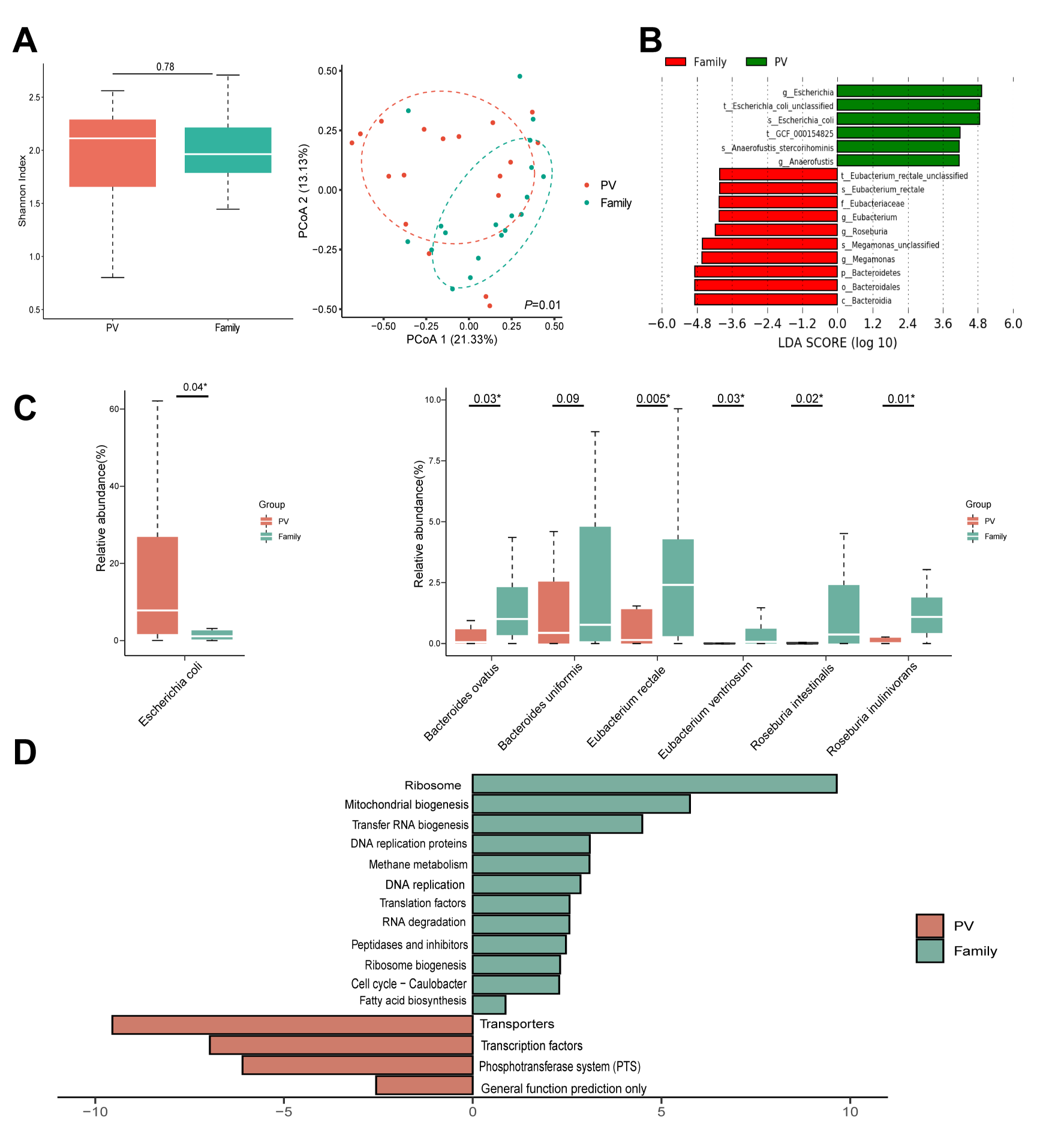

Supplement: Supplementary Figure 2 — Subgroup analysis specifically with PV and their matched family member controls. (A) The α-diversity (left) and β-diversity (right) of the gut microbiome in the PV patients and their matched family members; (B) Results of differential bacteria between PV and family members by LEFse analysis; (C) Main differential species between PV patients and their family members (* represents statistical significance with the P value <0.05 using Wilcoxon sum-rank test); (D) C-level difference KEGG pathway between PV patients and their matched family members, horizontal coordinate represents the P value of -log10. PV, pemphigus vulgaris; PCoA, principal coordinates analysis; LEFse, LDA effect size analysis; LDA, linear discriminant analysis; KEGG, Kyoto encyclopedia of genes and genomes. [file Image_2.tif]

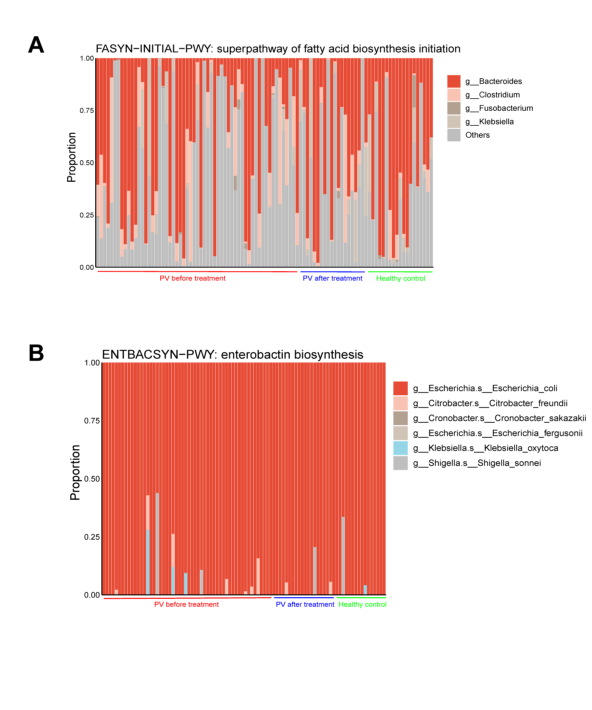

Supplement: Supplementary Figure 3 — Different MetaCyc pathways between PV patients and healthy controls. (A) Contribution of the microbial genus to the fatty acid biosynthesis pathway; (B) Contribution of the microbial species to the enterobactin biosynthesis pathway. [file Image_3.tif]
